# Perceived barriers to computerised quality documentation during anaesthesia: a survey of anaesthesia staff

**DOI:** 10.1186/1471-2253-15-13

**Published:** 2015-01-31

**Authors:** Johannes Wacker, Johann Steurer, Tanja Manser, Elke Leisinger, Reto Stocker, Georg Mols

**Affiliations:** Institute of Anaesthesia and Intensive Care, Hirslanden Clinic, Witellikerstrasse 40, CH-8032 Zürich, Switzerland; Horten Center for Patient-Oriented Research and Knowledge Transfer, University of Zurich, Pestalozzistrasse 24, CH-8091 Zürich, Switzerland; Institute for Patient Safety, Faculty of Medicine, University of Bonn, Stiftsplatz 12, D-53111 Bonn, Germany; Department of Anaesthesia & Surgical Intensive Care Medicine, Hospital Lahr-Ettenheim, Klostenstr. 19, D-77933 Lahr, Germany

**Keywords:** Anaesthesia, Attitude of health personnel, Mandatory reporting, Outcome and process assessment (Health Care), Quality assurance, Health care

## Abstract

**Background:**

Underreporting of intraoperative events in anaesthesia is well-known and compromises quality documentation. The reasons for such omissions remain unclear. We conducted a questionnaire-based survey of anaesthesia staff to explore perceived barriers to reliable documentation during anaesthesia.

**Methods:**

Participants anonymously completed a paper-based questionnaire. Predefined answers referred to potential barriers. Additional written comments were encouraged. Differences between physician and nurse anaesthetists were tested with t-tests and chi-square tests.

**Results:**

Twenty-five physician and 30 nurse anaesthetists (81% of total staff) completed the survey. The reported problems referred to three main categories: (I) potential influences related to working conditions and practices of data collection, such as premature entry of the data (indicated by 85% of the respondents), competing duties (87%), and interfering interruptions or noise (67%); (II) problems referring to institutional management of the data, for example lacking feedback on the results (95%) and lacking knowledge about what the data are used for (75%); (III) problems related to specific attitudes, e.g., considering these data not useful for quality improvement (47%). Physicians were more sceptical than nurses regarding the relevance of these data for quality and patient safety.

**Conclusions:**

The common perceived difficulties reported by physician and nurse anaesthetists resemble established barriers to incident reporting and may similarly act as barriers to quality documentation during anaesthesia. Further studies should investigate if these perceived obstacles have a causal impact on quality reporting in anaesthesia.

**Trial registration:**

ClinicalTrials.gov identifier is NCT01524484. Registration date: January 21, 2012.

**Electronic supplementary material:**

The online version of this article (doi:10.1186/1471-2253-15-13) contains supplementary material, which is available to authorized users.

## Background

Monitoring quality and safety is an important prerequisite for improving anaesthesia care [1–5]. Common techniques are reporting on quality indicators and on critical incidents, which depend on relevant and reliable data. Yet, incomplete documentation and underreporting of quality data in anaesthesia are well-known and occur even if computer-supported data collection is used [6, 7]. Available systems to gather such data [1, 4, 8] offer no generally accepted approach to verify the reliability of data.

Because we occasionally observed incomplete reporting on quality indicators during anaesthesia in our department, we conducted an analysis of a convenience spot sample of 50 cases. 65 well-defined intraoperative events (19 hypotensive, 31 bradycardic, 14 tachycardic, and 1 hypoxic events) were identified in the electronic anaesthesia records, but only 7 (11%) had also been correctly reported (unpublished observations: Wacker J, Manser T, Leisinger E, Stocker R, Mols G: Quality of Quality Data – a Pilot Study in Anesthesia. Conference Poster: International Conference “Patientensicherheit – avanti!”, Basel, Switzerland, 2011).

The conditions that specifically act as barriers to quality reporting are unclear so far. However, previous research has identified barriers to incident reporting (e.g., critical incident reporting systems, CIRS). Such barriers include fears of legal consequences, lacking knowledge and feedback about data and results, and unsatisfactory working conditions [5, 9–14]. Incident reporting on rare but potentially serious events has some parallels to quality reporting, but also differs significantly regarding background working conditions and techniques of data collection. Quality data are collected for every patient, typically as an additional routine task for busy anaesthesia staff during other clinical work. Moreover, research into barriers to incident reporting has also detected different reporting characteristics of physicians and nurses, for example more reluctance of physicians to report [10, 11, 15, 16]. From these various differences, the question arises if barriers similar to those reported for incident reporting may also affect quality reporting, and if such barriers relate to physicians and nurses in different ways.

This article presents the results of a survey in our department. By exploring the perspectives of experienced anaesthesia staff [17], we aimed at gaining an overview of perceived difficulties that may act as barriers to computerised quality reporting during anaesthesia, and of differences between professional groups. Preliminary data of this study have been presented as conference poster [18].

## Methods

### Ethics approval and license

The study was approved by the Ethics Committee of the Canton of Zurich, Switzerland (Reference Nr. KEK-ZH-Nr. 2011–0421). Written informed consent was obtained from all study participants. The ClinicalTrials.gov identifier is NCT01524484.

### Characteristics of the study hospital and anaesthesia department

The study was conducted at a major private hospital in Switzerland. Surgical activities comprise over 10’000 procedures in adult patients per year (2011/12), with orthopaedics, gynaecology/obstretrics, general surgery, visceral surgery, urology, neurosurgery, ENT, eye surgery, cardiac surgery, thoracic, and vascular surgery contributing most cases. Besides a large volume of elective intermediate and major surgical interventions, also emergency, minor, and outpatient procedures are performed using all current types of general and regional anaesthesia [19]. The anaesthesia care team typically consists of a board certified physician anaesthetist and a nurse anaesthetist or nursing student. Both are present during the periods of induction and emergence, and the physician is rapidly available throughout the entire case. During overlapping consecutive cases, physicians may supervise two nurse anaesthetists at the same time. Some anaesthetics, in particular challenging major cases, are entirely done by the physician with occasional nurse assistance. No resident physicians were part of the anaesthesia care team during the study period.

### Anaesthesia records and quality reporting at the study site

**Figure 1:**
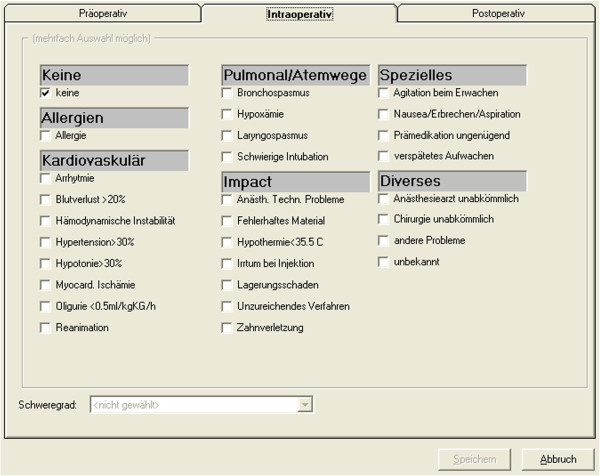
**Window for entry of intraoperative quality data into the anaesthesia information management system (screenshot).** Labeling and definition of event items are in german. “Präoperativ“, preoperative. “Intraoperativ“, intraoperative. “Postoperativ“, Postoperative. “Schweregrad“, severity (severity classification of the event). “Speichern“, save button. “Abbruch“, cancel button. Event categories: “Keine“, no events (clicked). “Allergien“, allergies. “Kardiovaskulär“, cardiovascular / heamodynamic events. “Pulmonal/Atemwege“, pulmonary and airway-related events. “Impact“, collective designation for various events, e.g. hypothermia, dental injury, and others. “Spezielles“, specific anaesthesia-related events, e.g. agitation upon emergence, nausea, and others. “Diverses“, organisational and other events.

At the study hospital, a custom-made anaesthesia information management system (AIMS) is used for both the electronic anaesthesia record charting and for quality reporting (Anästhesie Protokollierungs-System AP2011, © 1999–2011 Rolf Dinkelmann IFAI Hirslanden, Zürich; © 1998–2009 Dinkelmann Data Inform, Seestrasse 63, CH-8800 Thalwil, Switzerland). Computer workstations are part of each anaesthesia workplace. Data on preoperative risk factors and on a set of intraoperative anaesthesia quality indicators are manually entered by the end of each anaesthetic, gathering the necessary information from medical records, from memory (e.g., problems with airway management), and by checking the electronic anaesthesia record for deviations of the vital parameters. The system does not offer automated recordings of such deviations. According to departmental standards, quality data can be collected by both physician and nurse anaesthetists. In daily practice, these data are usually entered by the staff person, physician or nurse, providing most of the anaesthetic. To enter data, windows for quality data entry can be actively opened during the case via a task bar of the electronic anaesthesia record program in the AIMS. The windows display a selection of preoperative risks (e.g., cardiovascular conditions) and of intraoperative events/problems (e.g., hypoxaemia), which can be selected by clicking the respective box (see Figure 1). Information about event definitions is provided as hyperlinks when moving the cursor over the labels next to the boxes. An optional window for data collection on postoperative events/problems has not been activated for use in the department.By all means, data entry must be completed by the end of each case before transfer to the recovery room. To ensure capture of all quality-defining events of the anaesthetic including emergence and extubation in general anaesthetics, this should not be completed earlier or before extubation. The program obliges users to enter quality data before finalising the record by directing them to the quality windows following any attempt to close the anaesthesia record. Quality data entries into these windows are thus integrated as mandatory steps into the workflow of the electronic anaesthesia record, and as a minimum, the boxes for “no preoperative risk” and “no intraoperative event”, respectively, must be clicked (see Figure 1).

The indicators and definitions implemented in the AIMS meet the specifications of the Anaesthesia Databank Switzerland (ADS) anaesthesia registry [20, 21] (provided by IUMSP - Institut universitaire de médecine sociale et préventive, Unité d’évaluation des soins, 10, Route de la Corniche, CH-1010 Lausanne, Switzerland, and supported at the time of the study for voluntary participation of anaesthesia institutions by the Swiss Society of Anaesthesiology and Reanimation SGAR, CH-3000, Berne 25, Switzerland). Digitalized quality data are regularly forwarded from the anaesthesia department of the study hospital to IUMSP, which provides the department with annual quality reports.

### Questionnaire development

As to our knowledge, no validated survey instrument specifically measuring perceived barriers to quality reporting during anaesthesia was available at the time of our study. A questionnaire was therefore developed by the study team. A first set of questions was drafted by one author (JW) according to relevant topics taken from the literature on barriers to incident reporting. Questions focussed therefore on the following topics: User friendliness of the data collection system [9, 11], clearness of item definitions [9, 12, 14], beliefs about the effectiveness of quality reporting [9–11, 14], fears of legal consequences [9, 11, 14], existing or lacking knowledge about what the data are used for [9], if feedback about data and results was available [9, 11, 14], unsatisfactory working conditions such as lack of time [9, 11] and additional workload [9, 11]. With reference to the spot sample analysis described in the background section, which was not communicated to departmental staff before the survey, an estimate of current correct documentation of events during anaesthesia (hypotension, hypertension, bradycardia, tachycardia, hypoxia) and using the AIMS was additionally requested from the respondents.

The wording of the questions was customized to the anaesthesia working environment of the study site by the drafting author, who is familiar with this setting and is a practicing anaesthesiologist. The questionnaire was revised by two other authors with backgrounds in psychology and internal medicine regarding clearness of question content, wording, comprehensibility, and structure of the questionnaire. The third version was then revised by an additional anaesthesiologist regarding technical content and comprehensibility and re-checked regarding the survey objectives. The resulting revised version was circulated again among all authors. Three additional iterations were dedicated to the improvement of wording and clarity of the questions. As pilot test, the questionnaire was reviewed by anaesthesia staff of the study site not involved with the development of the questions. Final consensus yielded a 20-item, paper-based questionnaire offering predefined answer choices and inviting participants to add free-written comments.

The major subject areas covered by the questionnaire initially followed the topics taken from publications about barriers to incident reporting (see above). By customizing the questionnaire to the characteristics of the quality data collection process of the study site, the range of subjects was further modified. The questions included into the final version prior to data collection could be assigned a priori to three major subject areas: 1. Topics related to working conditions and practices of data collection; 2. topics related to the knowledge of in-house management of data on anaesthesia quality; 3. topics related to knowledge and to attitudes regarding data collection. These areas were used to summarize and present the results see results section (Tables 1, 2, 3).

**Table 1:** **Reported working conditions and practices of data collection**

Topic, Survey Questions (Italics)	Answer choices	Physicians	Nurses	Total	Significance
		(N = 24)	(N = 28)	(N = 55)	χ 2-test
**User friendliness:** *“Please assess the user friendliness of the electronic anaesthesia record/data entry system (AIMS)”*	*“good”* or *“sufficient”*	22 (92%)	26 (96%)	51 (94%)	n/a
	*“unsatisfactory"*	2 (08%)	1 (04%)	3 (06%)	
	*Total respondents*	24	27	54	
**Clarity of definitions:** *“Are the definitions of events (definition text) in the window ”SGAR problems and complications/intraoperatively“ unambiguous?”*	*“good”* or *“sufficient”*	19 (79%)	18 (67%)	39 (72%)	n/a
	*“unsatisfactory”*	5 (21%)	9 (33%)	15 (28%)	
	*Total respondents*	24	27	54	
**Adequacy of time available for data entry:** *“Do you have enough time to enter these data?”*	*“always”* or *“usually”*	22 (92%)	24 (86%)	49 (89%)	n/a
	*“rarely”* or *“never”*	2 (08%)	4 (14%)	6 (11%)	
	*Total respondents*	24	28	55	
**Timing of data entry:** *“When do you usually enter the data?” (before or after end of anaesthetic)*	*“after end”*	3 (13%)	2 (07%)	5 (09%)	n/a
	*“before end”*	19 (79%)	25 (89%)	47 (85%)	
	*“both”*	2 (08%)	1 (04%)	3 (05%)	
	*Total respondents*	24	28	55	
**Concomitant important duties during data entry:** *“Do you have to carry out other important duties at the time of quality data entry?”*	*“always”* or *“usually”*	22 (92%)	24 (86%)	48 (87%)	n/a
	*“rarely”* or *“never”*	2 (08%)	4 (14%)	7 (13%)	
	*Total respondents*	24	28	55	
**Which duties**: *“If yes: which duties?” (multiple answers possible; see text for items)*	Specific answers	23 (96%)	24 (86%)	49 (89%)	**
	No answers	1 (04%)	4 (14%)	6 (11%)	
	*Total respondents*	24	28	55	
**Frequency of noise or interruptions during data entry:** *“Please estimate how often noise or interruptions interfere with data entry”*	*“always”* or *“usually”*	15 (63%)	20 (74%)	36 (67%)	n/a
	*“rarely”* or *“never”*	9 (38%)	7 (26%)	18 (33%)	
	*Total respondents*	24	27	54	
**Record check before data entry:** *“Do you regularly perform a check of the anaesthesia record before data entry?”*	*“always”* or *“usually”*	6 (26%)	18 (67%)	25 (47%)	0.017*
	*“rarely”* or *“never”*	17 (74%)	9 (33%)	28 (53%)	
	*Total respondents*	23	27	53	

(%) indicate column percentages related to total respondents of the respective group to this question. Note: Results of three respondents who did not indicate their profession were not included in the comparison of professional groups. n/a, chi-square (χ2) test “not applicable” because the requirements for sufficient accuracy were not met, see methods section. *, accuracy of chi-square statistic was borderline. **, open question and therefore not included in group comparison. AIMS, Anaesthesia Information Management System; SGAR, Swiss Society of Anaesthesiology and Reanimation. Combined answers (e.g., “always” or “usually”) have been bundled into an essentially positive and an essentially negative answer category after data collection to facilitate interpretation (see methods section).

**Table 2:** **Knowledge of in-house management of data on anaesthesia quality**

Topics, Survey Questions (Italics)	Answer choices	Physicians	Nurses	Total	Significance
		(N = 24)	(N = 28)	(N = 55)	χ 2-test
**Reliability of data:** *“How reliable are the collected data, in your opinion?”*	*“reliable”*	1 (04%)*	5 (19%)	7 (13%)	n/a
	*“moderate”*	8 (33%)	17 (63%)	27 (50%)	
	*“unreliable”*	15 (63%)	5 (19%)	20 (37%)	
	*Total respondents:*	24	27	54	
**Feedback about quality data:** *“Do you get feedback about the collected data on quality?”*	*“no”*	22 (92%)	27 (96%)	52 (95%)	n/a
	*“yes”*	2 (08%)	1 (04%)	3 (05%)	
	*Total respondents*	24	28	55	
**Use of the data:** *“What are the collected data used for?”*	*“I don’t know”*	19 (79%)	21 (75%)	41 (75%)	n/a
	*“I know”*	4 (17%)	5 (18%)	10 (18%)	
	no answer	1 (04%)	2 (07%)	4 (07%)	
	*Total respondents*	24	28	55	
**Handling of identified problems by leading board and management:** *“How do the institute”s leading board and the hospital management handle problems and flaws identified with the systematic registration of intraoperative events?”*	answers	23 (96%)	23 (82%)	49 (89%)	**
	no answer	1 (04%)	5 (18%)	6 (11%)	
	*Total respondents*	24	28	55	
**Who should enter the quality data:** *“In your opinion, who should ideally enter these data: anaesthesia physicians or anaesthesia nurses?”*	*“Physician”*	20 (83%)	17 (61%)	40 (73%)	0.155
	*“Nurse”*	1 (04%)	1 (04%)	2 (04%)	
	*“both”*	3 (13%)	10 (36%)	13 (24%)	
	*Total respondents*	24	28	55	
**Need to improve data collection:** *“In your opinion, is there a need to improve data collection?”*	*“yes”*	8 (33%)	17 (61%)	27 (49%)	0.108*
	*“I don't know”*	11 (46%)	9 (32%)	21 (38%)	
	*“no”*	5 (21%)	2 (07%)	7 (13%)	
	*Total respondents*	24	28	55	
**Estimate of correct documentation:** *“Please estimate the percentage of “events” as captured in the record (categories hypotension, hypertension, bradycardia, tachycardia, hypoxia) correctly documented using the window “intra-operative events”*	mean	23.3%	38.5%	31.7%	0.012 t-test^1)^
	SD	18.2%	23.3%	22.0%	
	lowest	1	7.5	1	
	highest	50	90	90	

(%) indicate column percentages related to total respondents of the respective group to this question. Note: Results of three respondents who did not indicate their profession were not included in the comparison of professional groups. n/a, chi-square (χ2) test “not applicable” because the requirements for sufficient accuracy were not met, see methods section. *, accuracy of chi-square statistic was borderline. **, open question and therefore not included in group comparison. ^1)^means of estimates were compared using t-test; CI of the difference 3.4, 27.0.

**Table 3:** **Knowledge and attitudes regarding data collection**

Topics, Survey Questions (Italics)	Answer choices	Physicians	Nurses	Total	Significance
		(N = 24)	(N = 28)	(N = 55)	χ 2-test
**Relevance of data for patient safety:** *“In your opinion, how relevant is the collection of these data for patient safety?”*	*“relevant”*	8 (33%)*	19 (68%)	28 (51%)	0.032
	*“I don’t know”*	6 (25%)	5 (18%)	12 (22%)	
	*“irrelevant”*	10 (42%)	4 (14%)	15 (27%)	
	*Total respondents:*	24	28	55	
**Potential of data to improve anaesthesia quality:** *“In your opinion, do these data generally allow for an improvement of anaesthesia quality?”*	*“yes”*	4 (17%)	13 (46%)	18 (33%)	0.002
	*“I don’t know”*	2 (08%)	8 (29%)	11 (20%)	
	*“no”*	18 (75%)	7 (25%)	26 (47%)	
	*Total respondents*	24	28	55	
**Concern of easier prosecution because of the reported event data:** *“Do you have concerns of being prosecuted more easily based on the reported intraoperative event data in case of liability?”*	*“yes”*	2 (08%)	7 (25%)	9 (16%)	n/a
	*“I don’t know”*	4 (17%)	6 (21%)	11 (20%)	
	*“no”*	18 (75%)	15 (54%)	35 (64%)	
	*Total respondents*	24	28	55	
**Trust in data anonymisation:** *“Do you trust anonymisation of the quality data?”*	*“yes”*	9 (38%)	18 (64%)	29 (53%)	0.156*
	*“I don’t know”*	12 (50%)	8 (29%)	21 (38%)	
	*“no”*	3 (13%)	2 (07%)	5 (09%)	
	*Total respondents*	24	28	55	

(%) indicate column percentages related to total respondents of the respective group to this question. Note: Results of three respondents who did not indicate their profession were not included in the comparison of professional groups. n/a, chi-square (χ2) test “not applicable” because the requirements for sufficient accuracy were not met, see methods section. *, accuracy of chi-square statistic was borderline.

The questionnaire was not further validated for the use in other institutions. Based on the following circumstances, we deemed the questionnaire sufficiently comprehensive and valid for the purposes of our study: First, analogous items were introduced into the questionnaire from the available literature on incident reporting. Second, the final version reflected the differentiated views of an interprofessional author team including (but not limited to) anaesthesiologists familiar with the system under scrutiny. Third, the draft versions were repeatedly revised by the authors over a time period of about four months, allowing team members to contribute modifications or amendments after further reflection. Fourth, the comprehensibility for the survey target group was checked using pilot testing by anaesthesia staff of the study site not involved with the questionnaire development. For the detailed questionnaire, please see Additional file 1.

### Survey participants and exclusion criteria

The survey was conducted between November 2011 and February 2012 in the hospital’s anaesthesia department. All nurse and physician anaesthetists regularly working with the AIMS were considered for participation. Eligible anaesthesia staff at the study hospital was enrolled if they consented to participate in the study. Exclusion criteria were active involvement with the study (e.g., participation in study design, data acquisition), being responsible for quality data management, not working regularly with the AIMS, being on long-term leave, and lack of informed consent. In addition, one newly employed physician anaesthetist had been working with the AIMS for only about two weeks and therefore felt insufficiently familiar with the system to participate.

### Survey procedure

The principal investigator (JW) contacted eligible staff members directly at the workplace or by phone. Eligible participants received a brief oral description of the survey and detailed written information about the aims and organisation of the study. After participants gave their written informed consent, the questionnaire was handed out with a blank envelope for anonymous return to the principal investigator.

### Data handling and analysis

Data from the hardcopy questionnaires were recorded using spreadsheet software. Basic data analysis and frequency calculations were done using Microsoft Excel**®** 14.2.3 (Microsoft Corporation, Redmond, WA, USA) for Apple Macintosh (Apple Inc., Cupertino, CA, USA). Statistical analysis was performed with IBM SPSS**®** Statistics 20.0 for Apple Macintosh (SPSS Inc., an IBM Company, Chicago, IL, USA).

Of the 20 survey items, 17 items with nominal or continuous numerical data were assessed for differences between answers of physicians and nurses. Three items of the questionnaire (no. 8, 12, and 17; see Additional file 1) are open questions inviting free-written comments. Due to statistical reasons, these items were not included into the quantitative comparison. t-tests for independent samples were used to compare means between professional groups (nurse and physician anaesthetists) for continuous numerical data, if data were normally distributed. Normal distribution within samples was assessed using the one-sample Kolmogorov-Smirnov test for normal distribution. If data were not normally distributed, two-sample Kolmogorov-Smirnov tests were used as non-parametrical tests for independent samples. To compare the distribution of nominal answer variables between professional groups, crosstabulations were obtained, and associations between variables were assessed using the chi-square (χ2) test. Chi-square tests were considered sufficiently accurate if expected cell counts were never less than one, and no more than 20% of them were less than five [22]. In few cases (3 of 20 questionnaire items) where cell counts were less than five in 33.3%, accuracy was considered borderline, and the results of asymptotic 2-sided significance of the test were commented accordingly in the tables. A p-value of 0.05 was considered statistically significant.

To make the main findings easier to interpret, answers to six questions in Table 1 (“User friendliness”, “Clarity of definitions”, “Adequacy of time available for data entry”, “Concomitant important duties during data entry”, “Frequency of noise or interruptions during data entry”, “Record check before data entry”) were bundled into a positive (e.g., “always” or “usually”) and negative answer category (e.g., “rarely” or “never”) after data collection. To avoid misinterpretation, we have retained the original answer options and inserted a respective comment into the footnotes of Table 1. Chi-square tests were performed in these cases with transformed new variables that combined the bundled values into one single value.

## Results

### Characteristics of survey participants

Of the department’s staff of 68, 12 fulfilled the exclusion criteria. One person did not return the questionnaire. Thus, 55 participants completed the survey (25 physicians, 30 nurses). They represented 81% of total and 93% of eligible staff of the department. Three respondents did not indicate their profession (nurse or physician) on the questionnaire and were thus not included in group-specific analyses. On some questionnaires, information was lacking regarding level of employment (percentage of full position), professional experience, and/or experience with the AIMS.

No significant difference was noted between professional groups regarding level of employment (physicians: mean 82.2%, standard deviation (SD) 17.3; nurses: mean 89.5%, SD 17.7; data were not normally distributed, and two-sample Kolmogorov-Smirnov was p = 0.112; nine respondents did not answer this item). Similarly, there was no statistically significant difference between professional groups regarding experience with the AIMS (physicians: mean 5.0 yrs, SD 4.2; nurses: mean 5.0 yrs, SD 4.8; 95% confidence interval (CI) of the difference −2.7, 2.7, p = 0.997; eight respondents did not answer this item). In contrast, physicians had significantly longer professional experience than nurses (physicians: mean 17.2 yrs, SD 5.0; nurses: mean 10.8 yrs, SD 8.4; 95% CI of the difference 2.1, 10.7, p = 0.005; 11 respondents did not answer this item).

### Reported working conditions and practices of data collection

As shown in Table 1, the majority of respondents perceived the electronic data entry system as sufficiently user friendly. Further, most respondents stated they have enough time to enter the data. However, they indicated to do this usually before the end of the anaesthetic (for practical reasons, in most cases of general anaesthesia before extubation, and hence potentially missing events during or after extubation), without checking the anaesthesia record before data entry according the majority of respondents, and while carrying out other important duties. According to the free-written comments, concomitant duties included predominantly practical anaesthesia work (83%) and some administrative tasks (16%). For details, see Additional file 2.

### Knowledge of in-house management of data on anaesthesia quality

Most respondents (75%) indicated that they did not know what the collected data are used for. Only 18% knew what happens to the collected quality data and commented on this (Table 2). Others commented they were not aware of efforts by the department’s management to handle problems identified with the collected data (92% of comments; see Additional file 2). The predominant view (73%; Table 2) that physicians should ideally enter the quality data was confirmed by many free-written comments. 75% of these comments supported the view that only physicians should be responsible for data entry; 5% of comments suggested that this task should be accomplished exclusively by nurses; and 20% favoured the current institutional standard of data entry by both professional groups. Five respondents commented they thought that physicians had a better knowledge of the patient and his/her history. Three also remarked they thought physicians were “responsible for data”. On the other hand, some comments also favoured entry by nurses (“more reliable”) or by both groups (“both are responsible”; “four eyes see more”). Suggestions for improvements of data collection included the possibility of later data entry (22% of comments) as well as improvements of the content (30%) and layout (11%) of the quality data window (see Additional file 2).

### Knowledge and attitudes regarding data collection

Detailed results are displayed in Table 3.

### Comparison of answers between physicians and nurses

Significant differences between physicians and nurses were found for four nominal and one numeric item among 17 items suitable for comparison out of the 20 survey items. Three items could not be included because they represented open questions. Values of asymptotic, two-tailed significance of chi-square test comparisons are presented in Tables 1, 2, 3, if crosstable characteristics were sufficiently accurate (see methods section). Physicians made significantly lower estimates of correct documentation of events during anaesthesia than nurses (see Table 2).

## Discussion

In this survey, anaesthesia staff reported perceived difficulties and reservations that have the potential to act as barriers to computerised documentation of quality data during anaesthesia. These difficulties and reservations can be summarized into three categories: 1) potentially compromising influences on the process of data collection and unfavourable working conditions during data entry; 2) inadequate in-house management of the data, such as a lack of instructions about the use of the data and feedback about results; 3) negative attitudes of staff members regarding the relevance of these data for patient safety and anaesthesia quality. Physicians were more negative than nurses regarding the relevance of these data.

To our knowledge, the reasons for underreporting of data on intraoperative anaesthesia quality have not been specifically studied so far. This investigation did not aim at establishing a correlation between self-reported difficulties and the effectiveness of the reporting process or the actual quality of reported data. Rather, we aimed at exploring the perspective of staff on the process of quality data collection during anaesthesia. As underreporting is well-known in both quality and incident reporting [6, 7], a qualitative understanding of perceived difficulties related to quality reporting may be helpful as such to generate hypotheses about likely barriers.

Research about barriers to incident reporting, which differs markedly from quality reporting with regard to typical working conditions during collection of data, provides general insights into possible obstacles. Such barriers include fears of legal consequences, lacking knowledge about what is done with collected data, lacking feedback on the results of data analysis, and unsatisfactory working conditions during data collection [5, 9–14, 23]. However, barriers to quality reporting may not just be inferred from barriers to incident reporting. Despite some similarities, incident reporting and quality reporting differ considerably with regard to organisational and practical aspects. *Incident reporting* is used sporadically to generate single reports about sentinel events that indicate rare but serious hazards [2, 9] (e.g., injection of a wrong drug) and uses a separate reporting systems (CIRS) with a confidential or anonymous reporting process [24]. The primary responsibility for the data is usually assigned to a risk management subunit independent of the organisational hierarchy [25] (e.g., quality management). In contrast, *quality reporting*, following the concepts of the Physician Quality Reporting System in the US [4], involves systematic collection of routine data according to established, rate-based quality indicators for every patient [2]. It is usually not fully confidential or anonymous, less time-consuming per case, and mostly devoid of the perception of concern typical for critical incidents. The primary responsibility for these data is usually held by the management of the anaesthesia department.

Despite these basic differences, our results describe perceived difficulties and potential obstacles to quality reporting in anaesthesia that are comparable to the barriers to incident reporting described in the literature. This is partly unexpected. In contrast to incident reporting, quality reporting is a routine task and should be expected to work easily and to be generally accepted. Yet the reported knowledge deficits, negative attitudes, and perceived lack of feedback do not appear to meet the demands of an established standard.

Some of our findings require particular consideration. Surprisingly, 53% of respondents reported not to check the anaesthesia record before data entry, 85% reported to complete entry before the end of the anaesthetic, and 87% to have other important duties during data entry. This findings conflict for several reasons with the answer of 89% to have usually or always enough time to enter the data. First, if the anaesthesia record is not checked before data entry, events that should be reported can be overlooked. Second and more important, a comprehensive assessment of intraoperative anaesthesia quality should include the entire period of emergence, as quality-defining events may then also occur [26–28]. However, such events systematically escape capture if data entry is routinely completed before the end of the anaesthetic. Third, the reported concomitant other duties may not necessarily constitute a barrier, but have a general potential of interfering with data entry. In view of these limitations, the predominant clear statement of having enough time for data entry needs further explanation. The clear answer to complete data entry before the end of the anaesthetic (85%) suggests that collecting data about the entire course of the anaesthetic is not generally seen as a priority. Hence, the concurrent answer to have enough time to enter the data rather means that most respondents had enough time to perform an *incomplete* quality data collection than that they actually had enough time for *appropriate* data collection. If the respondents in fact have enough time for appropriate data entry cannot be concluded from this survey. The answers may reflect knowledge gaps or also “workarounds” to cope with the time limits [29]. Our data preclude causal inferences; however, these answers are also in line with the statement of 75% of respondents not to know what the collected data are used for, and of 95% not to get feedback about the collected data.

We also observed significant differences between responses of physician and nurse anaesthetists. Physicians had longer professional experience, reported a more sceptical attitude towards the relevance of data collection, more frequently indicated to omit checks of the anaesthesia record before data entry, and made more pessimistic estimates of correct documentation than nurses. These findings are generally in line with findings about incident reporting. In clinical settings outside anaesthesia for instance, nurses were found to know more about reporting [11], to report more habitually [15], and to be less reluctant to incident reporting than physicians [16]. In the perioperative setting, the self-reported willingness to report also differed between physician anaesthetists and PACU nurses or OR nurses [10]. Physician and nurse anaesthetists typically share their work environment and their practical tasks to a larger extent than physicians and nurses in most other clinical settings, but they also have different educational backgrounds and professional and legal responsibilities for patients. Differences between physician and nurse anaesthetists should thus also be considered with regard to potential barriers to quality reporting.

Interestingly, both professional groups concordantly stated that quality data should ideally be entered by physicians (73% of all respondents). Obviously, the established standard at the study site of quality data entry by both physicians and nurses does not fully match the expectations and beliefs of staff. Uncertainty about whose responsibility it is to report has been described as a potential barrier to incident reporting [9, 11]. According to our findings, unclear or poorly accepted responsibility for data collection may also constitute a potential barrier to quality reporting.

In summary, our study has three main implications for the understanding of flawed quality reporting in anaesthesia. First, the reported difficulties relate to three major areas concerned with quality reporting: Working conditions and practices of data collection, institutional management of the data, and specific attitudes. Delimitation of specific areas of difficulties as viewed by anaesthesia staff may help to conceptualize topics of particular interest for future research on barriers to quality reporting. The wide range of these difficulties also suggests that previously recommended strategies to maintain reporting compliance such as imposing minimal additional workload by the system, giving relevant feedback, and maintaining an atmosphere of confidence [1, 5, 30–34] may not cover all possible problems (e.g., noise and interruptions; particular attitudes; different perceptions of physicians and nurses) and may be subject to different local conditions. Second, the reported difficulties are similar to established barriers to incident reporting. This is not self-evident, because incident reporting and quality reporting differ considerably. It will be important to ascertain if self-reported difficulties with quality reporting correlate with flawed actual data quality in the same way as they do in incident reporting [16]. Third, distinct differences between answers of physicians and nurses revealed that physicians were more sceptical than nurses regarding the relevance of these data for quality and patient safety. Interestingly, most respondents stated that physicians rather than nurses should ideally enter these data. Differences between professional groups with regard to attitudes and self-reported behaviours should be borne in mind with respect to future research into factors influencing quality reporting.

This survey has a number of limitations. First, the observational design precludes inferences about causal effects of the reported potential barriers on quality reporting. Second, other actual causes may have been confounded due to possible survey-related biases. Third, our survey results are from one institution and might not be generalisable indiscriminately to other anaesthesia departments. Fourth, reporting bias due to limited trust in study anonymisation may have influenced the answers. Fifth, the survey instrument was not validated beyond the process of questionnaire development. The findings of this study can therefore not be directly used for process improvement. However, these limitations do not appear to have impaired the qualitative relevance of our results.

Further research should examine if the attitudes and opinions observed in this survey correlate with the actual practice of quality data collection, and with data quality. Beyond that, subsequent studies should also investigate interventions to specifically improve deficient quality reporting. Clarification of the effectiveness to achieve this goal is particularly needed for interventions aiming at improved working conditions during data entry, at optimised in-house management of the data including educational efforts addressing the use of the data and feedback about results, and at modifying negative attitudes of staff members regarding the relevance of quality data. Future research should also consider that self-reported behaviours and attitudes differ between physician and nurse anaesthetists, and that interventions may have to be adapted to the specific features and needs of these professional groups.

## Conclusions

The difficulties reported by anaesthesia staff in this survey have the potential to act as barriers to quality reporting in anaesthesia. Such perceived difficulties include potentially compromising influences on the working conditions during data entry, deficient in-house management of the data, scant knowledge of staff about the use of the data, and negative attitudes regarding their relevance, particularly among physicians. They further resemble previously reported barriers to incident reporting. Our findings do not establish a causal impact of these difficulties. Rather, they provide a conceptual basis for future research into the causes for deficient quality reporting and into interventions for improving data quality.

## Authors’ information

JW, EL: Consultant Anaesthesiologists, Institute of Anaesthesia and Intensive Care, Hirslanden Clinic, Zurich, Switzerland. JS: Director, Horten Center for Patient-Oriented Research and Knowledge Transfer, and Professor of Internal Medicine, University of Zurich, Switzerland. TM: Professor for Industrial Psychology and Human Factors, Department of Psychology, University of Fribourg, Switzerland; TM is now Director, Institute of Patient Safety, Faculty of Medicine, University of Bonn, Germany. RS: Head, Institute of Anaesthesia and Intensive Care, Hirslanden Clinic, Zurich, and Professor of Surgical Intensive Care Medicine, University of Zurich, Switzerland. GM: Head, Department of Anaesthesia & Surgical Intensive Care Medicine, Hospital Lahr-Ettenheim, Lahr, and Professor of Anaesthesia and Critical Care Medicine, University of Freiburg im Breisgau, Germany.

## Electronic supplementary material

Additional file 1: **Survey questions.** (PDF 96 KB)

Additional file 2: **Details of free-written comments to study questions.** (PDF 137 KB)

## Abbreviations

CIRS: Critical incident reporting systems
AIMS: Anaesthesia information management system
SD: Standard deviation
CI: Confidence interval
ESA: European Society of Anaesthesiology
SGAR: Swiss society of anaesthesiology and reanimation.
