# Supplementary material for: Perceived barriers to computerised quality documentation during anaesthesia: a survey of anaesthesia staff
Source: BMC Anesthesiol. 2015 Jan 31;15:13. doi: 10.1186/1471-2253-15-13 (PMC4429922; doi:10.1186/1471-2253-15-13)
Supplement: Supplementary file 1 — Additional file 1: Survey questions. (PDF 96 KB) [file 12871_2014_367_MOESM1_ESM.pdf]

Appendix 1 Survey Questions.

Abbreviations: AIMS: Anaesthesia Information Management System; SGAR: Swiss Society of Anaesthesiology and Reanimation

Date:………… Physician anaesthetist ☐ / Nurse anaesthetist ☐ Professional experience in anaesthesia (years):…… Experience with AIMS (years):……… Level of Employment (percentage of full position):………..%

1. Please assess the user friendliness of the electronic anaesthesia record/data entry system (AIMS)	good ☐ sufficient ☐ unsatisfactory ☐
2. Are the definitions of events (definition text) in the window "SGAR problems and complications/intraoperatively" unambiguous?	good ☐ sufficient ☐ unsatisfactory ☐
3. In your opinion, how relevant is the collection of these data for patient safety?	relevant ☐ I don't know ☐ irrelevant ☐
4. In your opinion, do these data generally allow for an improvement of anaesthesia quality?	yes ☐ I don't know ☐ no ☐
5. How reliable are the collected data, in your opinion?	reliable ☐ moderate ☐ unreliable ☐
6. Do you get feedback about the collected data on quality?	no ☐ yes ☐ If yes: How?...
7. What are the collected data used for?	I don't know ☐ I know! ☐ used for (short note): ....
8. How do the institute's leading board and the hospital management handle problems and flaws identified with the systematic registration of intraoperative events?	……………………………………………………………………… ………………………………………………
9. Do you have enough time to enter these data?	always ☐ usually ☐ rarely ☐ never ☐
10. When do you usually enter the data?	after end of anaesthetic ☐ before end of anaesthetic ☐
11. Do you have to carry out other important duties at the time of quality data entry?	always ☐ usually ☐ rarely ☐ never ☐
12. If yes: Which duties?	……………………………………………
13. Please estimate how often noise or interruptions interfere with data entry	always ☐ usually ☐ rarely ☐ never ☐
14. In your opinion, who should ideally enter these data: anaesthesia physicians or anaesthesia nurses?	Physician ☐ Nurse ☐ Reason:…………………….
15. Do you regularly perform a check of the anaesthesia record before data entry?	always ☐ usually ☐ rarely ☐ never ☐
16. In your opinion, is there a need to improve data collection?	yes ☐ I don't know ☐ no ☐
17. If yes: Do you have suggestions for improvements?	……………………………………………..
18. Do you have concerns of being prosecuted more easily based on the reported intraoperative event data in case of liability?	yes ☐ I don't know ☐ no ☐
19. Do you trust the anonymisation of the quality data?	yes ☐ I don't know ☐ no ☐
20. Please estimate the percentage of "events" as captured in the record (categories hypotension, hypertension, bradycardia, tachycardia, hypoxia) correctly documented using the window "intraoperative events"	………………………%
