# Supplementary material for: Perceived barriers to computerised quality documentation during anaesthesia: a survey of anaesthesia staff
Source: BMC Anesthesiol. 2015 Jan 31;15:13. doi: 10.1186/1471-2253-15-13 (PMC4429922; doi:10.1186/1471-2253-15-13)
Supplement: Supplementary file 2 — Additional file 2: Details of free-written comments to study questions. (PDF 137 KB) [file 12871_2014_367_MOESM2_ESM.pdf]

Appendix 2 Details of free-written comments to study questions.

Reported conditions and practices of data collection.

Question 11/12: *"Do you have to carry out other important duties at the time of quality data entry? If yes: which duties?"*

Of the respondents, 48 (87%) answered this question with "always" or "usually". The 84 related free-written comments referred to the following classes of important duties (numbers indicate frequency of respective comments; multiple comments were possible): *Administrative duties: total 13 (16%):* Documentation of the case: "anaesthesia record keeping" (4), "medical patient record" (1). Other documentation: "general paperwork" (1), "other data collection" (3), "other documentation" (1). Physician's medical duties: "preanaesthetic visits" (1), "prescriptions" (1). Others: answer pager calls (1). *Practical anaesthesia work: total 70 (83%):* "(administration of) anaesthetic" (38), "clearing of anaesthesia material" (2), "patient care" (7), "patient supervision" (10), "preparations for next case" (5), "termination of anaesthetic" (3), "patient positioning" (1), "patient transfer" (3), "assure patient safety" (1). *Unspecified other duties: total 1 (1%).*

Knowledge of in-house management of data on anaesthesia quality.

Abbreviations: AIMS: Anaesthesia Information Management System; CIRS: Critical Incident Reporting System; SGAR: Swiss Society of Anaesthesiology and Reanimation; AMDS: Absolute Minimal Data Set (SGAR Database)

Question 7: *"What are the collected data used for?"*

Of the respondents, 41 (75%) indicated they did not know what the collected data were used for, and 10 (18%) stated they did. Their 12 free-written comments (multiple answers possible) indicated the following uses for the data (numbers indicate frequency of respective comments): "SGAR" or "AMDS" (abbreviations: see text) (5), "statistics" (3), "survey" or "evaluation" or "benchmark" (4). Single remarks, which could not be clearly interpreted in context, comprised "little information about patient record", "CIRS", "optimisation of time schedule", "for next anaesthetic", "further monitoring".

Question 8: *"How do the institute's leading board and the hospital management handle problems and flaws identified with the systematic registration of intraoperative events?"*

To this question, 49 respondents (89%) made 49 specific free-written comments regarding this question (multiple comments possible) comprising (numbers indicate frequency of respective comments): *statements indicating no apparent activities or no awareness of such: total 45 (92%):* "I don't know" (16), "?" (12), "no feedback" (4), "no idea!" (2), "no information" (2), "they take note" (2), "not at all" (2), "very variably" (1), "no consequences" (1), "abortively" (1), "no systematic discussion" (1), "is this documented?" (1). *Statements indicating activities: total 4 (8%):* "information exchange within CIRS board" (1), "proactively!" (1), "solution is looked for" (1), "presentation to alert everybody" (1).

Question 14: *"In your opinion, who should ideally enter these data: anaesthesia physicians or anaesthesia nurses?"*

To this question, 40 respondents (73%) gave 40 different free-written comments (numbers indicate frequency of respective comments; multiple comments possible): *Comments supporting data entry by physicians: total 30 (75%):* "responsible for anaesthetic" (2), "responsible for data" (3), "general responsibility" (2), "better knowledge of patient & history" (5), "quality" (3), "assessment of severity" (2), "higher qualification, competence" (2), "has performed preoperative assessment" (1), "better assessment" (2), "medical knowledge" (1), "knowledge of risks & consequences" (2), "(data) interpretation" (2), "data intended for SGAR" (1), "longer presence" (1), "indication" (1). *Comments supporting data entry by nurses: total 2 (5%):* "more reliable" (1), "(closer) patient control" (1). *Comments supporting data entry by both groups: total 8 (20%):* "both are responsible" (1), "both experience complications" (1), "four eyes see more" (1), "whoever has given most of the anaesthetic" (2), "regardless; whoever has the time" (1), "both possible" (1), "depends on the situation" (1).

Question 16/17: *"In your opinion, is there a need to improve data collection? If yes: Do you have suggestions for improvements?"*

To this question, 21 respondents (38%) made the following 27 different specific free-written suggestions (numbers indicate frequency of respective comments; multiple comments possible): *Data analysis and staff information: total 2 (7%):* "regular analysis", "investigation of causes of the events". *Assignment of responsibility: 1 (4%):* "verification (of quality data) by postanaesthesia care unit". *General operability: total 2 (7%):* "handling"; "uncomplicated handling". *Content of quality data window: total 8 (30%):* "more accurate definition", "to be included: change of anaesthesia technique", "the quality data items (in the window) should contain defined criteria", "better questions (quality data items)", "legend", "more sophisticated", "differentiation according to clinical (situation)"; "comprehensive data collection desirable". *Layout of quality data window: total 3 (11%):* "better structuring", "free text field, more options for specifications", "free text for explanation". *Technical features of AIMS: total 2 (7%):* "it should not be possible to delete data", "automatic collection of, for example, haemodynamic data". *Timing of data collection: total 6 (22%):* "data entry at the end of the OR list", "data entry after end of anaesthetic", "data entry should be possible after closing the record", "data entry before anaesthetic (with text) and thereafter (complications) in later course", "intraoperative complications should be able to be captured at the end (of the case), as more relevant", "quality data window should still automatically appear (postoperatively) after record completion". *Working conditions: 1 (4%):* "more quiet during data entry". *Context not clear: 1 (4%):* "e.g., Dormicum 7.5 instead of 3.75 in premedication window". *Comment, but no suggestion for improvement: 1 (4%):* "await Narcodata (new AIMS, to be introduced at clinic)".
